# Disruption of gut homeostasis by opioids accelerates HIV disease progression

**DOI:** 10.3389/fmicb.2015.00643

**Published:** 2015-06-26

**Authors:** Jingjing Meng, Gregory M. Sindberg, Sabita Roy

**Affiliations:** ^1^Department of Surgery, Division of Infection, Inflammation, and Vascular Biology, Medical School, University of Minnesota, Minneapolis, MNUSA; ^2^Department of Veterinary Population Medicine, College of Veterinary Medicine, University of Minnesota, St. Paul, MNUSA; ^3^Department of Pharmacology, Medical School, University of Minnesota, Minneapolis, MNUSA

**Keywords:** opioids, HIV infection, disease progression, gut homeostasis, gastrointestinal immunology

## Abstract

Cumulative studies during the past 30 years have established the correlation between opioid abuse and human immunodeficiency virus (HIV) infection. Further studies also demonstrate that opioid addiction is associated with faster progression to AIDS in patients. Recently, it was revealed that disruption of gut homeostasis and subsequent microbial translocation play important roles in pathological activation of the immune system during HIV infection and contributes to accelerated disease progression. Similarly, opioids have been shown to modulate gut immunity and induce gut bacterial translocation. This review will explore the mechanisms by which opioids accelerate HIV disease progression by disrupting gut homeostasis. Better understanding of these mechanisms will facilitate the search for new therapeutic interventions to treat HIV infection especially in opioid abusing population.

## Introduction

Substantial epidemiologic studies report a greater risk of human immunodeficiency virus (HIV) infection in opioid addicts (Nath et al., 2002). Although confounding factors like widespread use of contaminated needles and nutritional status can contribute to the increased susceptibility of drug abusers to HIV infection, both clinical and laboratory studies provide strong evidence that opioid use alone increases risk of HIV infection by modulating the host’s immune function, enhancing pathogen entry into the immune cells and promoting virus proliferation (Roy et al., 2011). Opioid users experience more severe neurocognitive disorders than non-drug users, which are associated with higher levels of systemic inflammation (Bell et al., 1998, 2006; Anthony et al., 2008). However, the clear mechanisms underlying the complex interactions between opioids abuse and HIV infection still remain largely elusive.

Recent studies focusing on the gastrointestinal (GI) immune system illustrate that the gut associated lymphoid tissue (GALT) is the primary target of HIV during all stages of infection (Lackner et al., 2009) and the immunomodulatory effects of opioids on GI immune system may have consequences in gut defense mechanism and contribute to accelerated disease progression (Khansari et al., 2013). This review will summarize the mechanisms by which HIV and opioids modulate GI immunity and disrupt gut homeostasis, thereby driving pathological immune activation and disease progression following HIV infection.

## Overview of Gastrointestinal (GI) Immune System

The GI tract is considered the largest immunologic organ in the body and plays an important role in maintaining host immune homeostasis. Maintaining gut homeostasis requires balanced interactions among specialized epithelial cells, specialized lymphoid tissues, and commensal bacteria (Mason et al., 2008).

### (A) The Structure of Gut Epithelium

**FIGURE 1:**
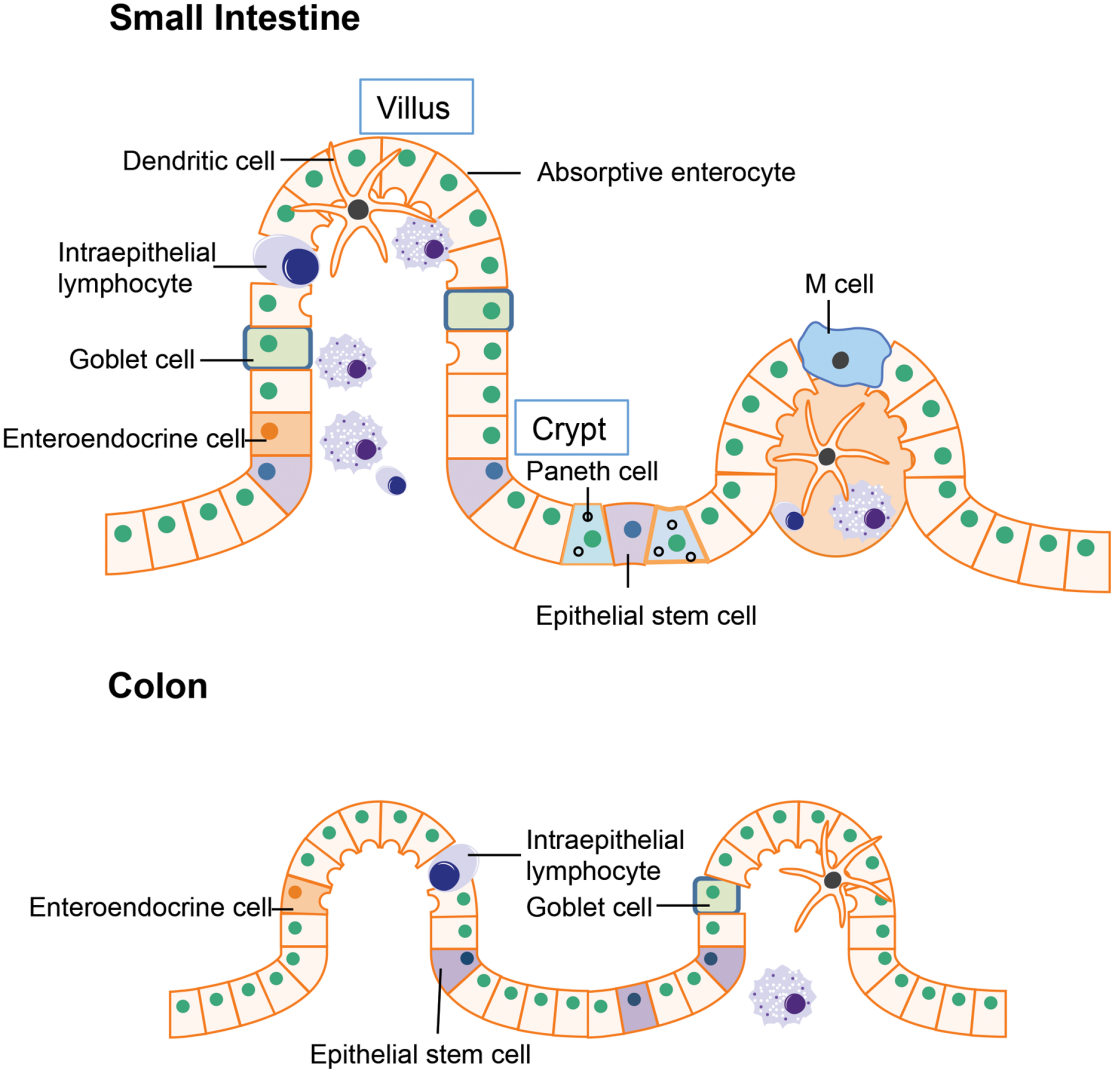
**Structure of gut epithelium.** In the small intestine, the epithelial stem cells located in the crypt differentiate into four cell types: the absorptive enterocytes, enteroendocrine cells, goblet cells, and paneth cells. In the colon, the epithelial stem cells are usually located in the lower parts of gut crypts and the differentiated cells migrate to the higher position and populate the colonic epithelial surface.

The intestinal epithelium is the first line of defense in the gut luminal environment. The well-organized intestinal epithelial cells not only provide physical barrier preventing potential pathogen or antigen invasion, but also is integral for nutrient support and water transport thereby maintaining homeostasis of the whole organism (Marchiando et al., 2010). The small intestine is where most chemical digestion and absorption of most of the nutrients from ingested food takes place. Besides digestion and absorption, the small intestine is also responsible for immune surveillance and defense against invading pathogens. To fulfill these multiple functions, the multi-potent stem cells located in the crypt region differentiates into four unique epithelial cell types: (i) absorptive enterocytes, (ii) enteroendocrine cells, (iii) goblet cells, and (iv) paneth cells (**Figure 1**; Bullen et al., 2006; Barker et al., 2007). Absorptive enterocytes and enteroendocrine cells in the villus are responsible for digestion and absorption. The goblet cells will migrate to the villus from crypt after differentiation and produce large amounts of mucins, which is the major component of the mucus layer lining the intestinal epithelium that provides additional protection to the epithelial barrier (Linden et al., 2008). Paneth cell stays in the base of the crypt after differentiation and is the major source of anti-microbial peptides such as α-defensins. The α-defensins are cysteine-rich cationic peptides with antimicrobial activity against a wide range of bacteria and other microbes. Studies of transgenic and knockout mice have supported a pivotal role of Paneth cell derived α-defensins for protection against bacterial pathogens (Porter et al., 2002). Another specialized epithelial cell residing in the small intestine is the M cell (or microfold cell). M cells are found in the follicle-associated epithelium overlaying the peyer’s patches (PPs). Unlike other intestinal epithelial cells, there is no mucus covering M cells, thus it plays a unique role in antigen presentation (Mabbott et al., 2013). In addition to the epithelial cells that have differentiated from crypt epithelial stem cells, certain immune cells express tight junctions proteins similarly to epithelial cells, allowing them to enter between epithelial cells. These include the dendritic cells and intraepithelial lymphocytes. In the intestines, dendritic cells can penetrate the epithelium to actively sample the antigens from the mucus layer (Rescigno and Di Sabatino, 2009). The intraepithelial lymphocytes are mainly involved in insuring the integrity of gut epithelium and maintaining the balance between normal immune responses and excessive inflammation (Cheroutre, 2005).

The major function of the colon is reabsorption of water and any remaining soluble nutrients from the food. The lack of villi in the colon results in a much smaller surface area compared to the small intestine. Organizationally, the epithelial stem cells are located in the lower parts of gut crypts and the differentiated cells migrate to the apical position and populate the colonic epithelial surface (Barker et al., 2007).

To avoid para-cellular leak of bacteria from gut lumen, the intercellular junctions known as tight junctions join adjacent cells together by extending from the cytoskeleton proteins of one cell into the extracellular matrix, finally joining with the tight junction proteins of another cell (Anderson and Van Itallie, 2009). Tight junction proteins are able to allow the transport of small solutes, water, and some macromolecules through the para-cellular pathway selectively and exclude the potentially harmful molecules. Tight junction proteins in the intestinal epithelium include transmembrane proteins belonging to the occludin and claudin families. These proteins seal the para-cellular pathway between the epithelial cells. In addition to transmembrane molecules, the para-cellular proteins include zona occludens-1 (ZO-1) and zona occludens-2 (ZO-2). They play an important role in supporting the organization of tight junction proteins (Peterson and Artis, 2014). The permeability of tight junction is dynamic and determined by the isoforms, quantity, and organizations of these proteins. Disruption of gut tight junction protein results in barrier defects and is associated with various intestinal diseases. For example, the genetic polymorphisms of the tight junction protein have been linked to celiac disease, ulcerative colitis, and Crohn’s disease (Schulzke et al., 2009).

### (B) Immune Cells in GALT, Mesenteric Lymph Node (MLN), and Lamina Propria

**FIGURE 2:**
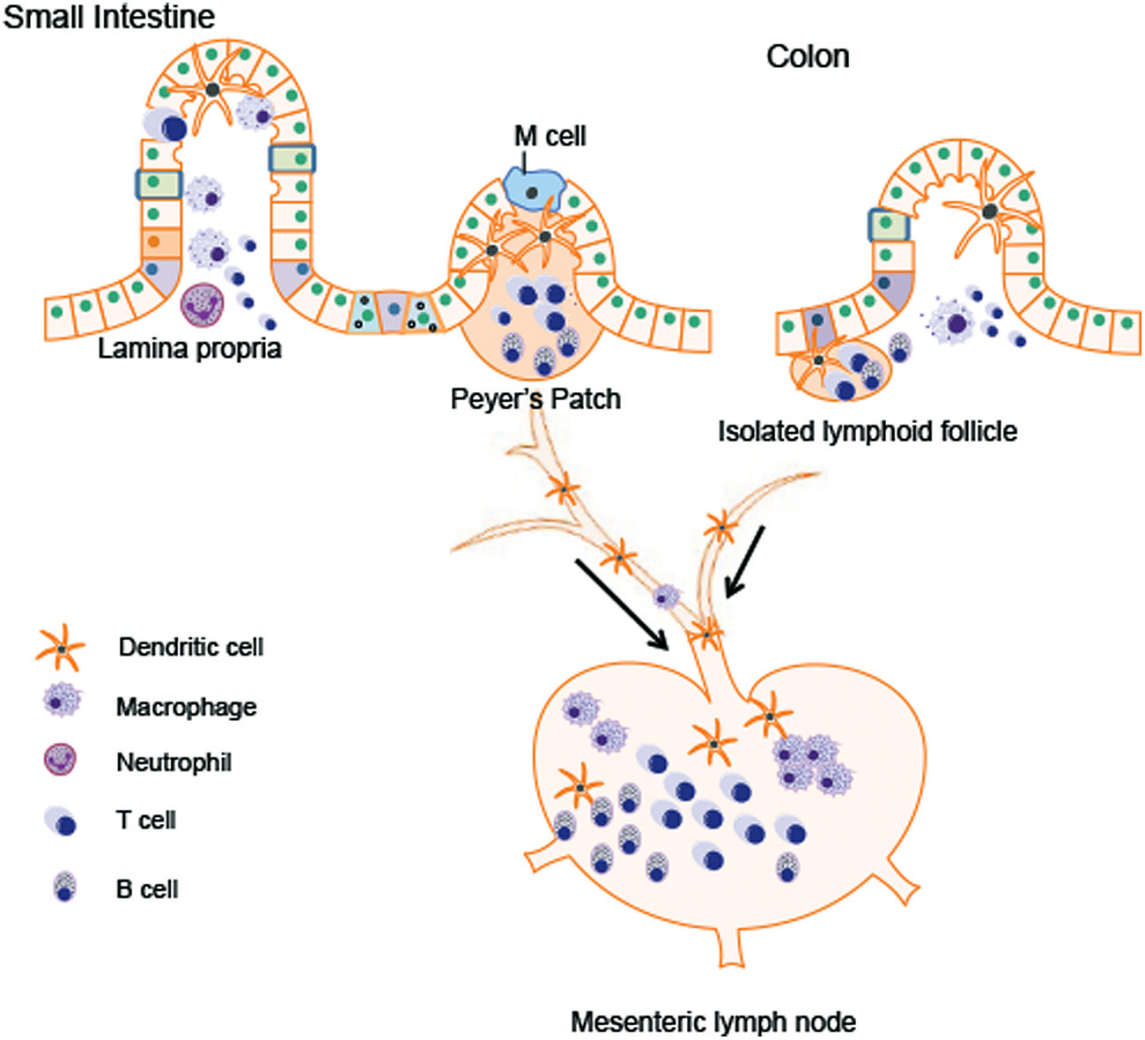
**Immune cells in gastrointestinal (GI) tract.** The major immune cell population within gut tissues includes macrophages, dendritic cells, neutrophilic granulocyte, T lymphocytes, and B lymphocytes. The Peyer’s patches in the small intestine and the isolated lymphoid follicle in the colon are composed of dendritic cells, B cells and T cells, which are crucial for antigen presentation from gut lumen. Antigen presenting cells (APCs) migrate to the mesenteric lymph node (MLN) through afferent lymphatic system. By receiving the signals presented by APCs, MLN amplify the immune responses. Lamina propria T cells have the characteristics of activated effector lymphocytes. The major populations of T cells residing in lamina propria are the CD4^+^ T cells, predominated by Th1 and Th17 population.

Different types of immune cells are distributed in GALT like PPs and isolated lymphoid follicles, mesenteric lymph node (MLN), and the lamina propria. The major immune cell population within gut tissues includes macrophages, dendritic cells, mucosal mast cells, neutrophilic granulocyte, eosinophilic granulocyte, T lymphocytes, and plasma cells (**Figure 2**). These cells can modulate the intestinal microenvironment by secreting antibodies, cytokines, or chemokines as well as mediate interaction with the enteric nervous system (ENS).

In both humans and rodents, PPs are organized lymphoid nodules usually found in the ileum and less frequently in the jejunum. PPs are usually covered by follicle-associated epithelium, which contains special M cell. M cell can sample antigen directly from the gut lumen and deliver it to antigen-presenting cells like dendritic cells. T cells, B cells, and memory cells in PPs are stimulated upon encountering antigen. These cells migrate to the MLN through afferent lymphatic system. By receiving the signals presented by dendritic cells, MLN amplify the immune responses. In this way, MLN plays a key role in tolerance induction to food proteins and in host defense against the pathogens from the gut lumen (Mason et al., 2008). PPs are not present in the colon. Isolated lymphoid follicles provide immune surveillance and protection against potential pathogen instead of PPs (Koboziev et al., 2010; Suzuki et al., 2010; Lycke and Bemark, 2012).

Within the lamina propria, immune cells constantly monitor for abnormalities both in the intestinal lumen as well as inside host GI tissue. Antigen presenting cells (APCs) includes dendritic cells, macrophages, and neutrophils. These cells recognize antigens from the gut lumen and present them to adaptive lymphocytes (T cells and B cells) to initiate the immune responses. Various cytokines and chemokines produced by these APCs are also involved in the regulation of intestinal inflammation (Farache et al., 2013). Most lamina propria T cells have the characteristics of activated effector lymphocytes. The major populations of these T cells are the CD4^+^ T Cells, predominated by Th1 and Th17 population. Th1 cells produce IFN-γ, a cytokine important for the control of virus infection and responsible for the pathogenesis of inflammatory bowel disease. Th17 cells produce Th17 cytokines including interleukin (IL)-17A, IL-17F, and IL-22 (Shale et al., 2013). The role of Th17 cytokines in host defense against extracellular pathogens will be discussed in the following part. IgA^+^ plasma cells are the dominant population in the lamina propria. The secretory IgA produced by plasma cells can neutralize toxins and mediate ingestion of pathogen via opsonization (Suzuki et al., 2010).

Taken together, both adaptive and innate immune cells are collectively important for maintaining homeostasis within the GI tract. Disruption of any cellular component may potentially predispose hosts to infections or initiate an abnormal immune response that accelerates disease progression.

### (C) Gut Microbiota in Gut Immunity

Gut commensal microbiota is an essential compartment of the gut immune system playing a critical role in maintaining intestinal homeostasis. The gut microbiome is responsible for the breakdown of complex nutrients that the host cannot digest and metabolize toxic xenobiotic agents into harmless metabolite (Tremaroli and Bäckhed, 2012). They are also able to exclude potential pathogens in the gut lumen (Sekirov et al., 2010). Secondly, they are involved in formation of mucus layer outside the epithelium. Previous studies have shown that microbial flora influences the number of goblet cells, their mucin production, and the glycosylation of mucins (Gaskins, 2001). Moreover, they are essential for the development of lymphoid tissues like PPs and are able to modulate T cell and B cell responses (Lee and Mazmanian, 2010). For example, most commensal bacteria have no direct contact with intestinal epithelial cells except for the segmented filamentous bacteria (SFB), which are able to adhere to epithelial cells especially to those in the PPs. Interestingly, recent studies show that the mice without SFB have significantly less IL-17 production in their intestines, implying the important role of SFB in Th17 differentiation (Ivanov et al., 2009). Not surprisingly, lack of gut commensal microbiota will result in abnormalities such as increased susceptibility to infectious diseases. For example, germ-free mice, which lack microbial colonization, show increased susceptibility to *Salmonella* due to impaired IgA production and diminished T cell response (Hapfelmeier et al., 2010). The germ-free mice also lack inflammatory CD4^+^ T-helper cells as well as regulatory T cells (Tregs; Strauch et al., 2005; Niess et al., 2008).

## HIV-Mediated Disruption of Gut Homeostasis

The mucosal tissues are primary sites of viral transmission and the major sites for viral replication and CD4^+^ T cell destruction. With the largest mucosal surface, the GI tract is targeted during all stages of HIV infection and plays a central role in the pathogenesis of AIDS.

### (A) Compromised GI Epithelial Barrier Function and AIDS Pathogenesis

Cumulative studies have demonstrated that HIV infection exerts a negative impact on intestinal structure and function that begins early in infection and might favor disease progression via microbial translocation and generalized immune activation.

One of the mechanisms contributing to compromised intestinal epithelial integrity is the deficiency of epitheliotropic factors, which are required for epithelial cell growth, maintenance, and renewal. Elevated levels of apoptosis-related gene expression like caspase-3 are observed in epithelial cells while genes involved in epithelial differentiation pathways like casein kinase 2A1 and Wnt 5A are down-regulated during primary HIV infection (Sankaran et al., 2008), supporting that the maintenance and renewal mechanisms of gut epithelial cells are impaired following HIV infection.

Human immunodeficiency virus has also been shown to modulate the tight junctions between both endothelial and epithelial cells (Mahajan et al., 2008; Estes et al., 2010). HIV has been shown to modulate tight junctions by disrupting CD4^+^ T cells, which typically support the maintenance of tight junctions (Shanahan, 1999). However, studies have also shown that HIV proteins such as Tat (transactivator of transcription) and GP120 can directly disrupt tight junctions on epithelial cells in culture (Bai et al., 2008; Nazli et al., 2010). This may be the mechanism in early pathogenesis for how bacterial translocation gets initiated to start a vicious cycle of immune activation and infection in the GI tract. Further studies indicate that increases in proinflammatory cytokine production in the intestines may be involved in tight junction modulation as well. Up-regulation of proinflammatory cytokine in the colon as early as 2.5 days following SIV infection (Hirao et al., 2014) and in the intestine of HIV-infected patients (McGowan et al., 2004; Sankaran et al., 2005) could facilitate mucosal damage by activating myosin light chain kinase (MLCK; Turner, 2006).

As discussed above, the epithelial barrier in the GI tract is crucial in maintaining a sterile barrier between the lumen of the intestine and the host. The central role of gut epithelial barrier in the disease progression following SIV infection has been investigated using African green monkeys and the sooty mangabey (Gordon et al., 2007; Pandrea et al., 2007; Silvestri et al., 2007; Klatt et al., 2012) Despite high viral loads there is no disease progression to AIDS in these animals although the infection induces a rapid loss of intestinal CD4^+^ T cells, which is similar to what has been observed in HIV-infected humans and SIV-infected macaques. In contrast to the persistent immune activation in HIV-infected humans and SIV-infected macaques, the attenuated immune activation may explain the non-progressive SIV infection in African Non-human Primates. Moreover, the immune activation is usually associated with compromised GI integrity and subsequent microbial translocation (Klatt et al., 2010). All these data suggest that the microbial translocation from the leaky gut plays a determinant role in disease progression following SIV infection.

### (B) Immune Cells in Disease Progression During HIV Infection

Recent studies demonstrate that CCR5^+^CD4^+^ memory T cells are primary targets for HIV/SIV (Okoye and Picker, 2013). And the gut lamina propria contains a much larger percentage of mucosal CCR5^+^CD4^+^ memory T cells compared to peripheral lymphoid tissues, which may explain why the GI tract is a major site of CD4^+^ T cell depletion and viral replication in HIV/SIV infection (Zeitz et al., 1988; Veazey et al., 1998). HIV/SIV infection causes rapid depletion of CCR5^+^CD4^+^ T cells by 21 days after infection and most infected cells in the intestine are present in immune inductive sites (Veazey et al., 1998, 2000, 2001). The early loss of CD4^+^ T cells in the gut following HIV/SIV infection contributes to the disruption in normal intestinal epithelial barrier function as discussed in the previous section, resulting in systemic translocation of bacterial products (Brenchley et al., 2006).

Specifically, CD4^+^ Th17 cells have been shown to play an important role in mediating the pathogenesis of AIDS following infection, as studies comparing pathogenic and non-pathogenic SIV infection have shown a preferential loss of these cells in pathogenic infections (Brenchley et al., 2008; Cecchinato et al., 2008; Peng et al., 2013). Th17 cells produce IL-17 and IL-22 instead of interferon gamma or IL-4. These cells play a unique role in maintaining intestinal homeostasis and production of antimicrobial defensins (Monteleone et al., 2010; Rubino et al., 2012; Gu et al., 2013). Therefore, the loss of Th17 cell in gut may provide a potential mechanism for gut barrier dysfunction and microbial translocation.

Following microbial translocation, some of the microbial products have been shown to activate immune cells, leading to localized cytokine production and activation of other immune cells such as CD4^+^ T cells thereby providing new substrates for HIV/SIV replication. The proinflammatory cytokine produced by the activated immune cells further compromise the intestinal epithelial barrier function. For example, IL-1β produced by the paneth cells in the crypt is responsible for gut barrier disruption following SIV infection (Hirao et al., 2014). Besides promoting gut barrier dysfunction and intestinal inflammation, the proinflammatory cytokine IL-6 can also promote viral replication. A recent study by Mohan et al. (2008) show that IL-6 induces a significant increase in the expression levels of CCAAT/Enhancer Binding Protein (C/EBP)-β following SIV infection. C/EBP-β binds to the long terminal repeat (LTR) of HIV and SIV in the infected cells of gut lamia propria, facilitating both viral replication and intestinal inflammation (Mohan et al., 2008).

### (C) HIV-Induced Dysbiosis in the Gut Microbiome

More recently, evidence suggests that disease progression following HIV/SIV infection is associated with dysbiosis in the microbiome of infected patients at multiple sites. In areas where opportunistic infections take place, such as the lingual cavity, the microbiome has been suggested to become more pathogenic (Dang et al., 2012). Within the GI tract, microbial populations such as proteobacteria species, *Prevotella*, and Enterobacteriaceae have been shown to be correlated with increase immune activation and increased in abundance in HIV patients (Dillon et al., 2014; Mutlu et al., 2014). Interestingly, species of Proteobacteria have been identified in the brains of HIV-infected patients (Branton et al., 2013), which is likely a cumulative result of microbial dysbiosis and disruption of tight junctions especially in the blood brain barrier. Certain strains have also been correlated with better health in HIV patients. For instance, high abundance of Lactobacillales have been shown to be protective of CD4^+^ T cell loss and microbial translocation (Pérez-Santiago et al., 2013). Metabolic changes have also been correlated to microbial dysbiosis (Vujkovic-Cvijin et al., 2013). These changes are likely a result of prolonged immune activation characteristic of HIV patients and exacerbate dysfunction within the GI tract. Additionally, most recent study using SIV models suggest that the administration of antiretrovirals induces substantial microbiome dysbiosis in the GI tract while virus alone shows only modest effects on the gut microbiome (Klase et al., 2015). Therefore, more studies are needed to better understand how HIV and/or antiviral therapy modulate gut microbiota and how dysbiosis in the gut microbiome correlate with infection and contribute to the pathogenesis of AIDS.

## GI Disorders Associated with Opioid Abuse

### (A) Opioid Receptor Distribution in Gut

Opioids exerts their actions by binding to several subtypes of opioid receptors, including (i) μ-receptors, (ii) δ-receptors, (iii) κ-receptors, and (iv) non-classical opioid receptors (Waldhoer et al., 2004). Originally, opioid receptors were thought to be expressed only in the central nervous system. However, a wealth of literature now provides evidence that opioid receptors are also expressed in peripheral tissues such as the GI tract. Several groups have attempted to characterize opioid receptor expression in peripheral tissues using quantitative real-time RT-PCR. Their results showed that all μ-, δ-, and κ-receptors have low expression levels in the small intestine (Peng et al., 2012). Using a similar approach in rats further validated the occurrence of μ-, δ-, and κ-receptors in both small and large intestine (Wittert et al., 1996). In gut tissues, opioid receptors are mainly expressed in the ENS, which is comprised of the myenteric and submucosal plexus. By binding to the opioid receptors in the ENS, various endogenous and exogenous opioids are able to modulate GI motility and secretion. Besides ENS, opioid receptors expressed on immune cells have been shown to play a role in intestinal inflammation (McCarthy et al., 2001; Philippe et al., 2003). Interestingly, it has been reported that enterocytes isolated from the crypt epithelium of guinea pigs have both μ- and δ- opioid receptors (Lang et al., 1996). Opioid receptor expression in intestinal epithelial cells is further supported by Rousseaux et al.’s (2007) study, in which they show that opioid receptors in intestinal epithelial cells could be up-regulated by lactobacillus, which may provide novel approaches for the treatment of abdominal pain.

**FIGURE 3:**
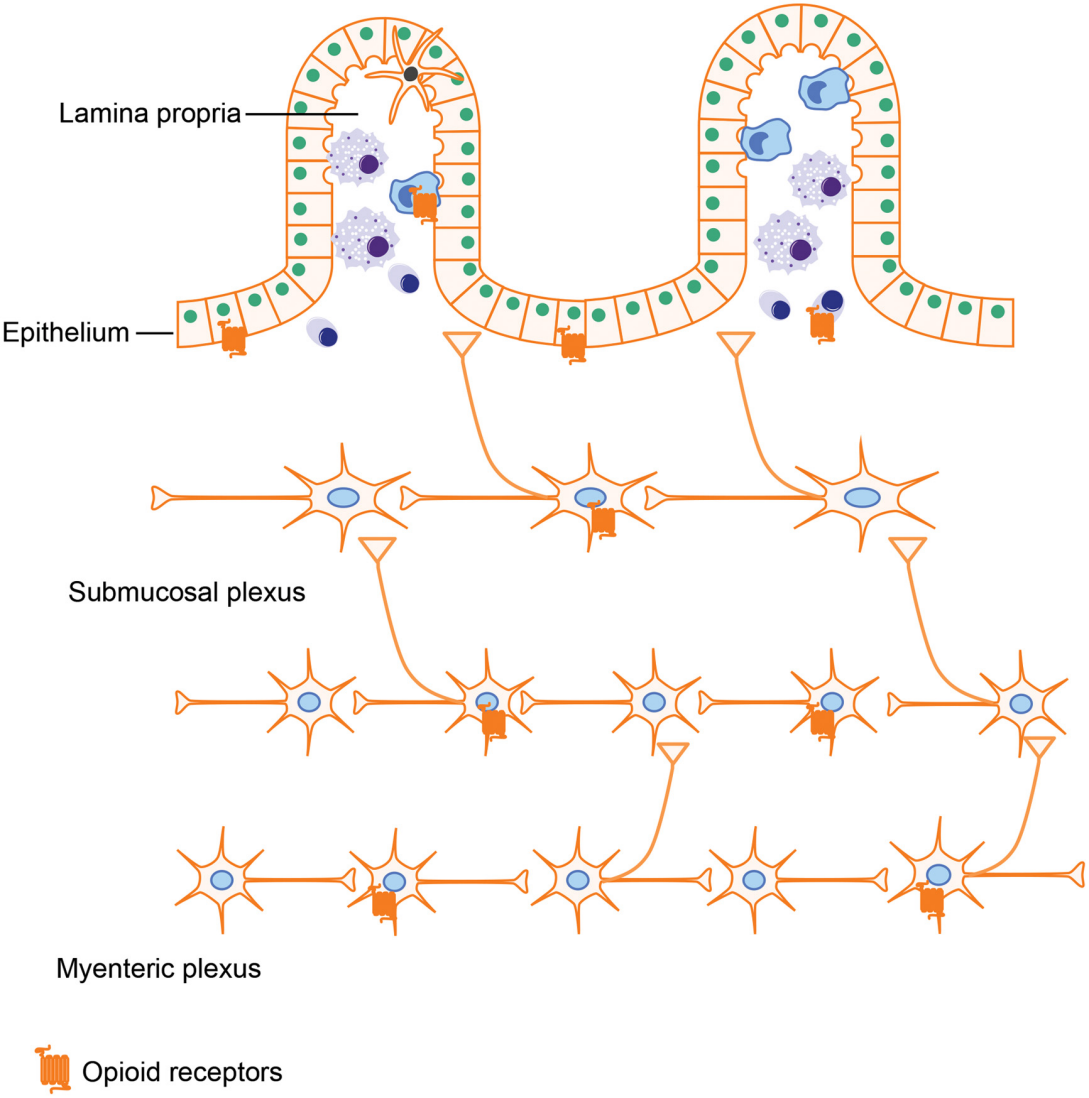
**Opioid receptor distribution in intestines.** Opioid receptors are not only expressed on neutrons in the myenteric plexus and submucosal plexus, which are involved in modulating gut motility and secretion but are also expressed on intestinal epithelial cells and immune cells, implying that opioids could impact the gut barrier function and immune responses as well.

By activating these opioid receptors in intestinal tissues (**Figure 3**), opioids exert both pharmacological and adverse effects in the gut, including alleviating abdominal pain, modulating GI motility, and suppressing intestinal immune functions.

### (B) Adverse GI Effects Associated with Opioid Use

**FIGURE 4:**
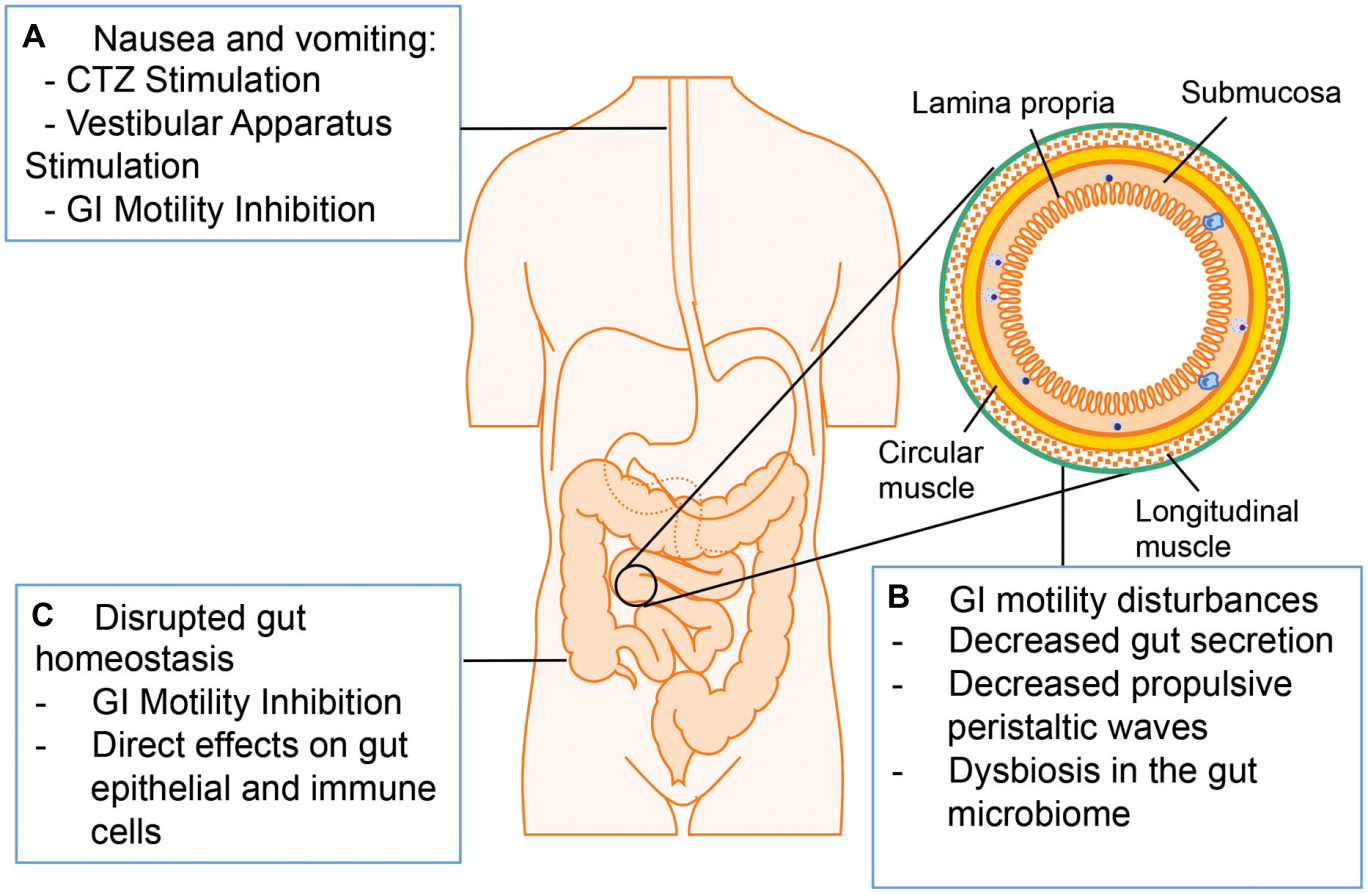
**Adverse GI effects associated with opioid administration. (A)** Opioid treatment induces nausea and vomiting via chemoreceptor trigger zone (CTZ) stimulation, vestibular stimulation, and GI motility inhibition. **(B)** Opioid treatment induces GI motility disturbances by decreasing propulsive peristaltic waves, inhibiting GI secretion and modulating gut microbiome. **(C)** Opioid treatment disrupts gut homeostasis by influencing gut epithelial cells and immune cells directly or by inhibiting GI motility.

The major adverse GI symptoms in opioid abuser include nausea, vomiting, constipation, and bloating (**Figure 4**; Miaskowski, 2009; Tuteja et al., 2010; Khansari et al., 2013). Nausea and vomiting are one of the common side effects associated with opioid analgesics. Multiple mechanisms are involved in opioid-induced nausea and vomiting, including direct stimulation of the chemoreceptor trigger zone (CTZ) for vomiting, inhibition of gut motility, and stimulation of the vestibular apparatus. Opioids are small molecules, which can cross blood–brain barrier and stimulate CTZ via the activation of μ- and δ-receptors. Opioid inhibition of gut motility results in gut distension, extended GI emptying time, and constipation, which stimulates visceral mechanoreceptors and chemoreceptors to induce nausea and vomiting. Other studies suggest that opioids bind to μ-receptors in the vestibular apparatus directly and the stimulation of vestibular apparatus results in nausea and vomiting (Porreca and Ossipov, 2009).

Another adverse symptom associated with opioid treatment is a decrease in GI motility. The normal motor functions of the GI tract are crucial for mixing and propelling food particles at rates that allow absorption of nutrients, cleaning the proximal intestine of residual food and bacteria, and enabling mass movement. Thus, the bowel dysfunction induced by opioid treatment may lead to serious consequences in patients (Ukleja, 2010). There are multiple mechanisms contributing to opioid-induced decreased GI motility: Opioids are able to interact with the opioid receptors on presynaptic nerve terminals in the myenteric plexus to initiate signals that increase intestinal resting tone to the point of spasm while decreasing propulsive peristaltic waves. Moreover, by binding to μ-receptors within the ENS, opioids treatment increase activity of the sympathetic nervous system and inhibit vasoactive intestinal peptide release thus affecting gut secretion and absorption. The overall decreased gut secretion causes a delay in digestion, increased water and sodium reabsorption and formation of dryer and harder stools, which can contribute to prolonged transit of the intestinal contents through the GI tract. There are two major types of GI motility disorder limiting the clinical application of opioids: postoperative ileus and constipation (Miaskowski, 2009). Constipation is the major GI motility disorder occurring with chronic opioid treatment. Although laxatives or opioid antagonists can alleviate constipation, their efficacy is still insufficient (Tuteja et al., 2010). The disrupted GI motility may contribute to increased susceptibility to infections with gut origin considering that impaired peristalsis results in accumulation of residual food and bacteria in the gut lumen. Clinical studies imply that the early enteral feeding could enhance immune function and decrease the risk of infections in postoperative patients, so delayed enteral feeding because of opioid treatment and subsequent ileus may be responsible for increased risk of infection. Additionally, the opioid loperamide has been shown to shift microbiome taxonomic and metabolic profile, and these changes contribute to development of constipation in mice (Touw et al., 2014). As discussed previously, the dysbiosis in the gut microbiome plays a crucial role in GI homeostasis disruption and promotes pathogenesis of gut infectious diseases especially for HIV infection. Thus it will be interesting to investigate how opioids and/or opioid-induced GI motility disorder influence gut microbiome and the physiological consequence of this modulation.

Despite the observation that opioid receptors are expressed on immune cells within the intestinal lamina propria, studies focusing on the effects of opioids on intestinal immune function and inflammation are relatively sparse. Researches using animal models show that both chronic morphine and morphine withdrawal can lower host defense to enteric bacteria such as *Salmonella enterica*, *Acinetobacter baumannii*, and *Pseudomonas aeruginosa*. Opioids can induce bacterial translocation into the systemic system resulting in sepsis in mice (Hilburger et al., 1997; Ocasio et al., 2004; Feng et al., 2006; Breslow et al., 2011; Babrowski et al., 2012; Meng et al., 2013). In addition to bacterial translocation, morphine has been documented to increase pro-inflammatory cytokine production in rats and accelerate the progression of LPS-induced sepsis to septic shock (Roy et al., 1999; Greeneltch et al., 2004; Ocasio et al., 2004). In clinical studies, higher circulating morphine levels were observed in patients with sepsis, severe sepsis, and septic shock compared with healthy controls, implying the role of opioids in sepsis progression (Glattard et al., 2010). Overall, both clinical and laboratory studies provide evidence that besides the indirect effects mediated by GI peristalsis, other direct effects of opioids on epithelial cells and immune cells on intestinal lamina propria and GALTs also play important roles in impaired gut immune function. However, the mechanisms underlying compromised gut immune function and increased susceptibility to infections after opioid treatment have not been well studied.

## Opioids Promotes HIV Progression by Disrupting Gut Homeostasis

Chronic opioid abuse is of particular public health concern for the spread of infectious diseases, especially in the context of the HIV epidemic. In certain regions, up to 40% of HIV infected individuals are believed to abuse opioids (UN Joint Programme on HIV/AIDS (UNAIDS), 2013). Opioid users also suffer from a faster progression of infection to AIDS, as well as severe long term consequences such as neurocognitive defects in both humans and non-human primate models studied (Kapadia et al., 2005; Rivera-Amill et al., 2010; Byrd et al., 2011). Studies using peripherally acting μ-opioid receptor antagonist methylnaltrexone indicate that opioids can increase the infectivity of cells and viremia, which are mediated by the peripheral μ-opioid receptors (Ho et al., 2003).

Most recent studies have focused on how opioid abuse promotes neurocognitive dysfunction like HIV-associated neurocognitive disorders (HAND) in the infected individuals. All these studies consistently showed that opioid abusers experience more severe neurocognitive defects including HIV-associated dementia (Bell et al., 1998, 2006). Further mechanistic studies correlate the increased neurotoxicity of HIV in opioid abusers with up-regulated inflammation (Gurwell et al., 2001; El-Hage et al., 2008; Bokhari et al., 2009).

**FIGURE 5:**
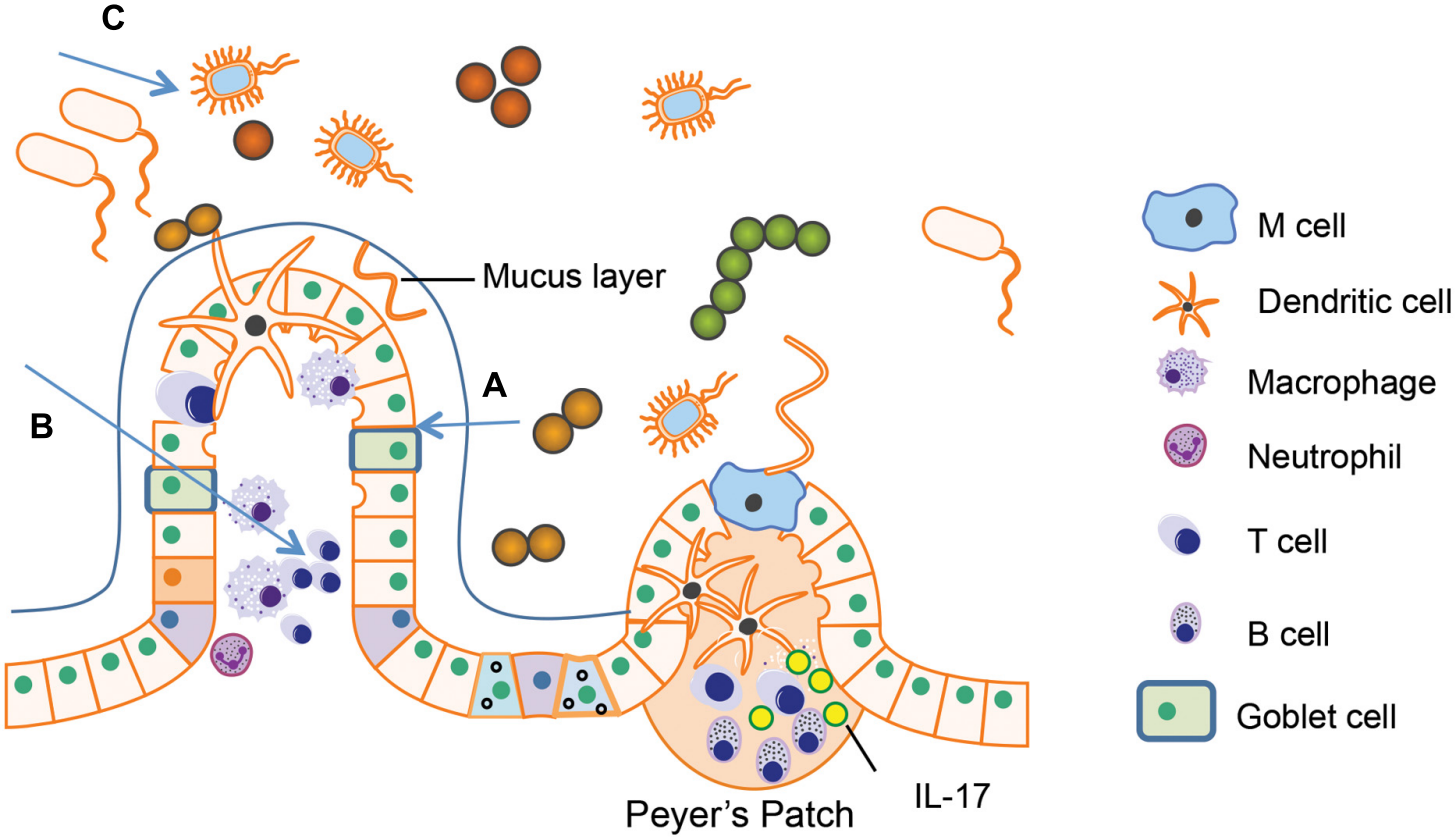
**Both human immunodeficiency virus (HIV) infection and opioid abuse induce disruption of gut homeostasis. (A)** Opioids and HIV infection can induce intestinal barrier disruption and subsequent microbial translocation. **(B)** Opioids and HIV infection can induce an abnormal inflammatory response in the gut. **(C)** Opioids and HIV infection has been shown to induce dysbiosis in the gut microbiome.

However, the effects of opioid abuse on GI homeostasis during HIV infection are not well studied although both opioids and HIV have been shown to disrupt the intestinal homeostasis through the similar pathways (**Figure 5**). As previously described, both HIV/SIV infection and morphine treatment can induce the intestinal epithelial barrier damage. Indeed, humans infected with HIV who use heroin have been shown to have greater amounts of LPS in their serum compared with non-users (Ancuta et al., 2008), implying that disruption of barrier function within the gut is more severe in drug using HIV patients. An investigation of gut biopsy from HIV-infected patients displays microtubule depolymerization, which is expected to be associated with intestinal tight junction disorganization and as a result of MLCK activation (Clayton et al., 2001; Turner, 2006). Interestingly, our study also shows that morphine treatment alone induces intestinal tight junction disruption and bacterial translocation by activating MLCK (Meng et al., 2013). Therefore, it is conceivable that opioids and HIV infection either synergistically or additively modulate MLCK activation explaining the more sever gut barrier dysfunction observed in drug abusing HIV infected patients. This was recently validated by our studies where it was demonstrated that opioids exacerbated HIV induced gut barrier disruption, increased bacterial translocation from the gut lumen, and sustained systemic inflammation with an increase in both IL-6 and TNF-α using a rodents model of HIV (Sindberg et al., 2014).

In addition to gut barrier damage, both HIV infection and opioid abuse have been shown to affect the functions of gut immune cells, resulting in abnormal immune activation in the gut and accelerated disease progression. Chronic morphine has been implicated to modulate populations and activities of Tregs and Th17 cells. The T cell populations with surface markers characteristic of gut-homing (CD161 and CCR6) and HIV-1 susceptibility (CCR5 and α4β7 integrin) were also increased in morphine-treated animals (Cornwell et al., 2013). Recent reports indicate that integrin α4β7 binds to HIV-1 gp120, together with CD4 and CCR5, forms a complex with the viral envelope glycoprotein, which promotes cell–cell spreading of HIV-1 (Piguet and Sattentau, 2004; Arthos et al., 2008). On basis of this data, it is reasonable to speculate that opioid abuse will enhance HIV replication and spread in the gut, which contributes to accelerated disease progression in opioid abuser.

## Conclusion

Cumulative studies focusing on gut homeostasis help us recognize the central role of gut mucosal tissues in the pathogenesis of AIDS following HIV infection. Virus infection can induce compromised gut epithelial barrier function, abnormal immune activation within the gut tissues, and the dysbiosis in the gut microbiome, which in turn facilitates HIV replication and the disease progression.

Additionally, more recent studies demonstrate that opioid abuse can disrupt gut homeostasis in the HIV-infected individuals. The severe disruption of gut homeostasis observed with opioids and models of HIV infection supports what is observed in the clinical studies as HIV patients who inject opioids have higher levels of bacterial translocation than patients who do not use opioids, which is likely why HIV patients who abuse opioids have more severe pathogenesis of HIV and develop AIDS much more quickly than non-users. However, the molecular mechanisms underlying the interactions of opioid abuse and HIV-induced enterotoxicity are still not well understood. Better understanding of these mechanisms may allow for therapeutic interventions to prevent or slow the pathogenesis in the gut following HIV infection, which will finally lead to more powerful strategy to control HIV disease progression especially in the opioid using and abusing population.

## Conflict of Interest Statement

The authors declare that the research was conducted in the absence of any commercial or financial relationships that could be construed as a potential conflict of interest.
